# Triple-stapled quadrilateral anastomosis: a new technique for creation of an esophagogastric anastomosis

**DOI:** 10.1007/s10388-017-0599-z

**Published:** 2017-12-16

**Authors:** Yuji Ishibashi, Tetsu Fukunaga, Shinya Mikami, Shinichi Oka, Satoshi Kanda, Yukinori Yube, Yoshinori Kohira, Takeharu Enomoto, Takehito Otsubo

**Affiliations:** 1 Department of Gastroenterology and Minimally Invasive Surgery, Juntendo University Hospital, Juntendo University School of Medicine, 3-1-3 Hongo, Bunkyo-ku, Tokyo, 113-8431 Japan; 2 Division of Gastroenterological and General Surgery, St. Marianna University School of Medicine, Kanagawa, Japan

**Keywords:** Esophagogastric anastomosis, Linear stapled anastomosis, Anastomotic leakage, Esophageal cancer

## Abstract

**Background:**

Esophagogastric anastomosis performed after esophagectomy is technically complex and often the source of postoperative complications. The best technique for this anastomosis remains a matter of debate. We describe a new all-stapled side-to-side anastomosis, which we refer to as triple-stapled quadrilateral anastomosis (TRIQ), that can be performed after minimally invasive surgery, and we report results of a retrospective evaluation of postoperative outcomes among the 60 patients in whom this anastomosis has been performed thus far.

**Methods:**

The anastomosis is created by apposition of the posterior walls of the esophagus and stomach. A linear stapler is applied to create a V-shaped posterior anastomotic wall. The anterior wall is closed in a gentle chevron-like shape with the use of 2 separate linear staplers, resulting in a wide quadrilateral anastomosis. The anastomosis is then wrapped with a greater omentum flap.

**Results:**

The patient group comprised 48 men and 12 women with a mean age of 67.8 years. Neoadjuvant chemotherapy was performed in 43 of these patients. Neither the thoracoscopic or laparoscopic procedure was converted to open surgery in any patient. The median operation time was 474 min (range 680–320 min). The intraoperative blood loss volume was 104.4 mL (range 240–30 mL). There were no anastomosis-related complications above Clavien-Dindo grade II.

**Conclusions:**

TRIQ can be performed easily and safely, and good short-term outcome can be expected.

## Introduction

Esophagectomy is routinely performed for esophageal carcinoma and for end-stage benign esophageal diseases. However, the esophagogastric anastomosis, whether circular stapled anastomosis, linear stapled anastomosis, or hand-sewn anastomosis, is technically complex and not without potential postoperative complications, such as anastomotic leakage and stricture. The incidence of anastomotic leakage, in particular, remains unacceptably high at a reported range of 10–13% [1–4]. Both short-term and long-term complications negatively affect the patient’s quality of life after esophagectomy. Use of a linear stapler for creation of the anastomosis has been described recently, and the techniques have been reported to reduce the rates of leakage and stricture associated with hand sewing techniques [5, 6]. The optimum technique remains a matter of debate. We developed an all-stapled side-to-side anastomosis, which we refer to as triple-stapled quadrilateral anastomosis (TRIQ) and have thus far applied in 60 patients treated by thoracoscopic esophagectomy for squamous cell carcinoma. We recently conducted a retrospective evaluation of the surgical safety and feasibility of TRIQ for creation of an esophagogastric anastomosis, and herein describe the procedure and results of our study.

## Materials and methods

### Patients and data collection

Between January 2014 and February 2017, a total of 60 patients underwent surgery for esophageal cancer at either Juntendo Hospital or St. Marianna University School of Medicine Hospital in Japan. All patients underwent endoscopy, an upper gastrointestinal series, and contrast-enhanced computed tomography preoperatively, and all were diagnosed with Stage 0, Stage I, Stage II, or Stage III disease for which esophagectomy is indicated [7]. Minimally invasive esophagectomy (thoracoscopic esophagectomy and laparoscopic gastric mobilization with abdominal lymph node dissection) and TRIQ were performed in all 60 patients. All of the operations were performed by the same surgeon (TF).

To assess the safety and outcomes of our TRIQ, we reviewed patient records for the following: patient characteristics, including age, sex, location of the tumor, and whether neoadjuvant chemotherapy was performed or not; duration of the operation; blood loss volume; postoperative hospital stay; postoperative complications, including anastomotic leakage and stricture; and pathological findings. Patients’ body temperature, pulse, C-reactive protein level, and white blood cell count had been monitored postoperatively for detection of anastomotic leakage; patients had also been checked for excess fluid at the cervical wound. Anastomotic leakage was identified on the basis of clinical signs. Anastomotic stricture was defined as any stricture in the anastomotic region that required endoscopic balloon dilatation. Complications were graded according to the Clavien-Dindo classification system as follows: Grade I, any deviation from the normal postoperative course without the need for pharmacological, surgical, endoscopic, or radiologic intervention but with allowance for antiemetics, antipyretics, analgesics, diuretics, electrolytes, and/or physiotherapy; Grade II, any complication requiring pharmacological treatment with drugs other than those allowed for Grade I complications and/or requiring blood transfusion or total parenteral nutrition; Grade IIIa, any complication requiring intervention not under general anesthesia; Grade IIIb, any complication requiring intervention under general anesthesia; Grade IVa, single organ dysfunction; Grade IVb, multiorgan dysfunction; Grade V, death [8].

### TRIQ technique

**Fig. 1:**
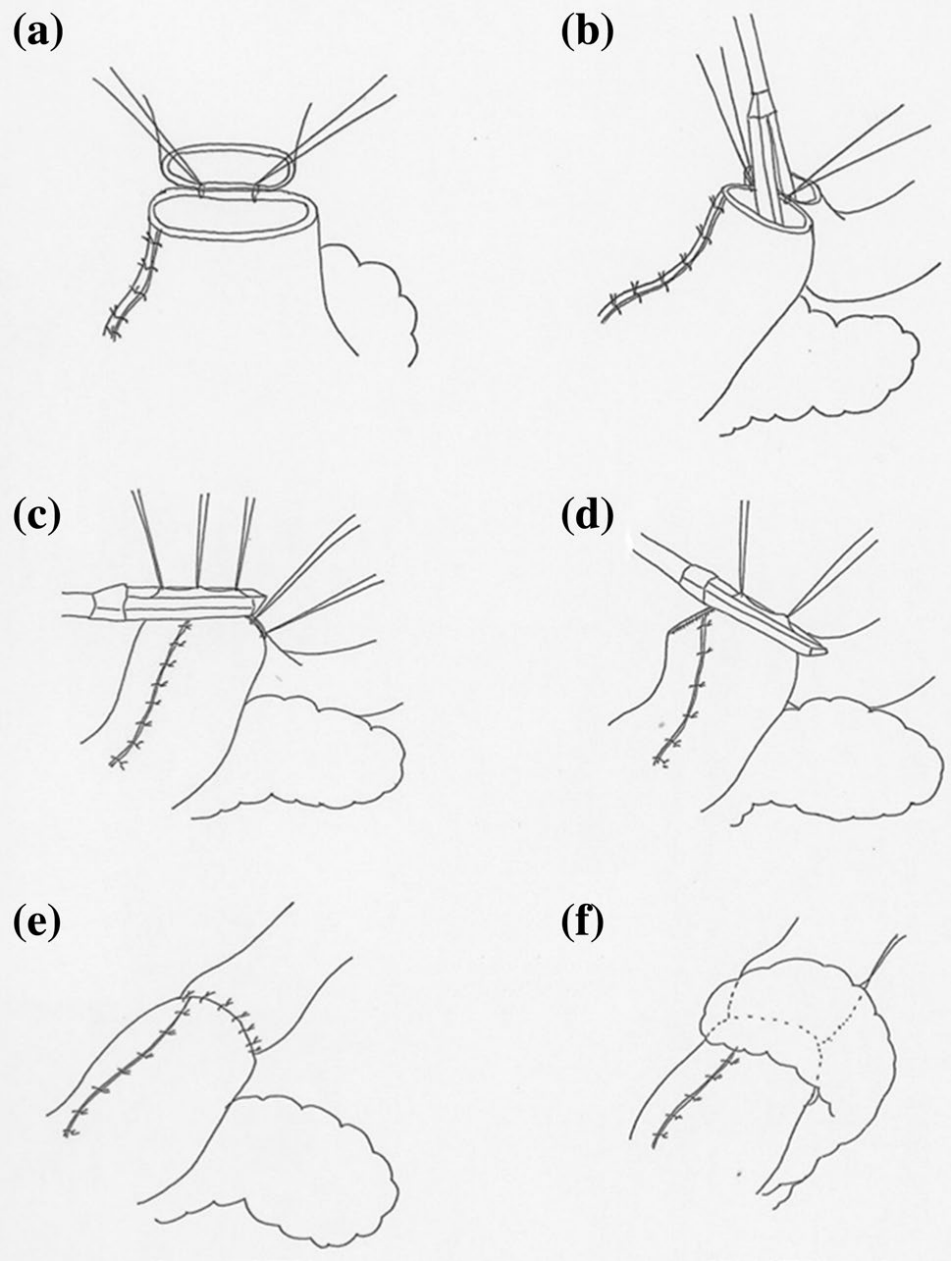
Initial steps in creation of the triple-stapled quadrilateral anastomosis. **a** With apposition of the posterior walls of the esophagus and stomach, 2 stitches are placed to anchor the esophageal and stomach wall. **b** A 60-mm linear stapler is applied between 2 stitches to construct the V-shape posterior wall of the anastomosis. **c**, **d** The anterior wall is closed in a gentle chevron-like shape with 2 60-mm linear staplers. **e** The anterior wall is closed with Lembert sutures to reinforce the anastomosis and avoid formation of a tracheal fistula. **f** The greater omentum flap is wrapped around the anastomosis

**Fig. 2:**
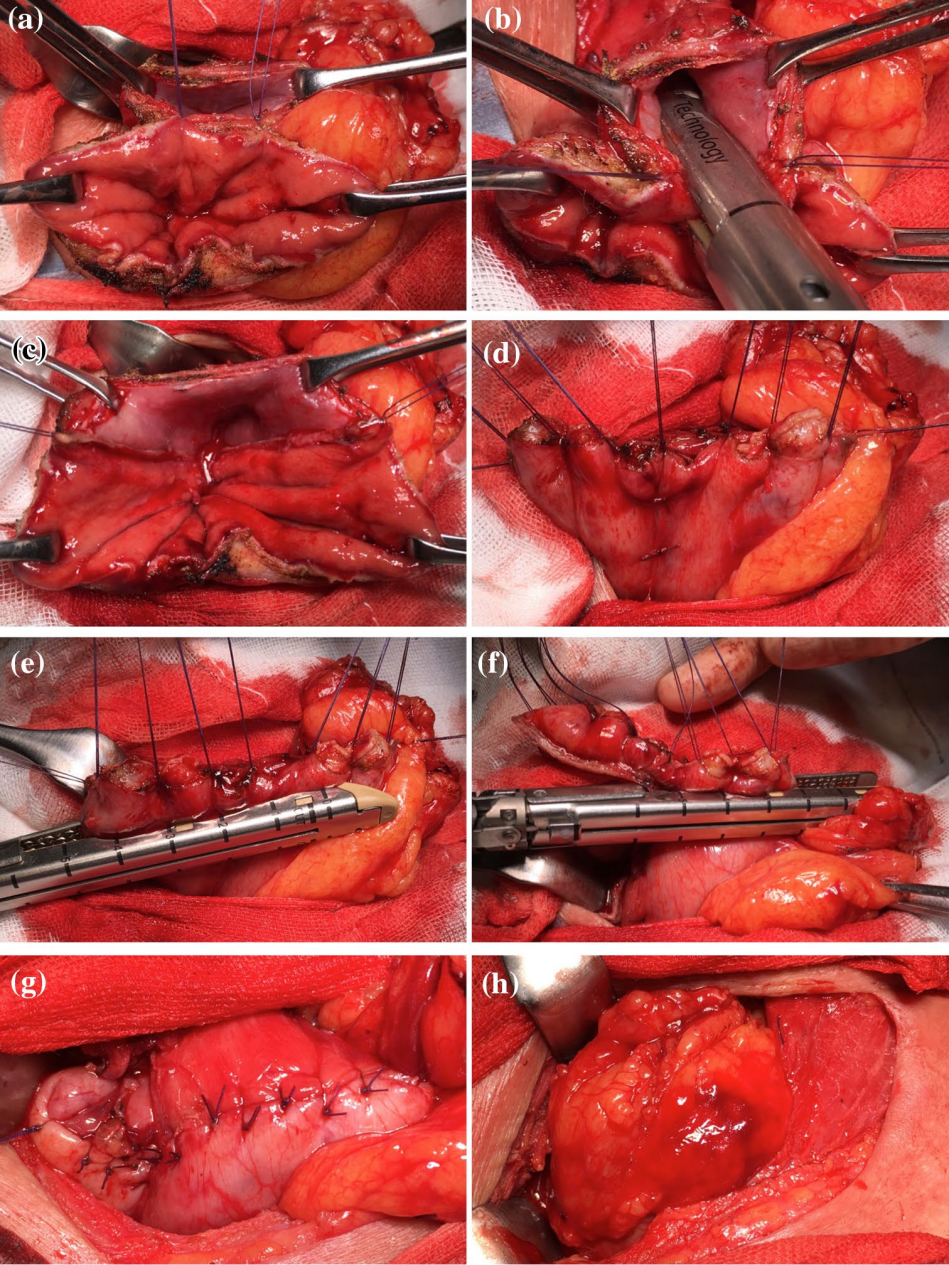
Intraoperative photographs showing completion of the triple-stapled quadrilateral anastomosis. **a** 2 stitches are placed to anchor the esophageal and stomach wall. **b** A linear stapler is applied between 2 stitches. **c** The V-shaped posterior wall of the anastomosis. **d** The anterior wall is everted and elevated with the use of additional supporting sutures. **e**, **f** The anterior wall is closed with 2 separate linear staplers. **g** The anterior wall is closed with Lembert sutures. **h** The anastomosis is wrapped with a greater omentum flap

**Fig. 3:**
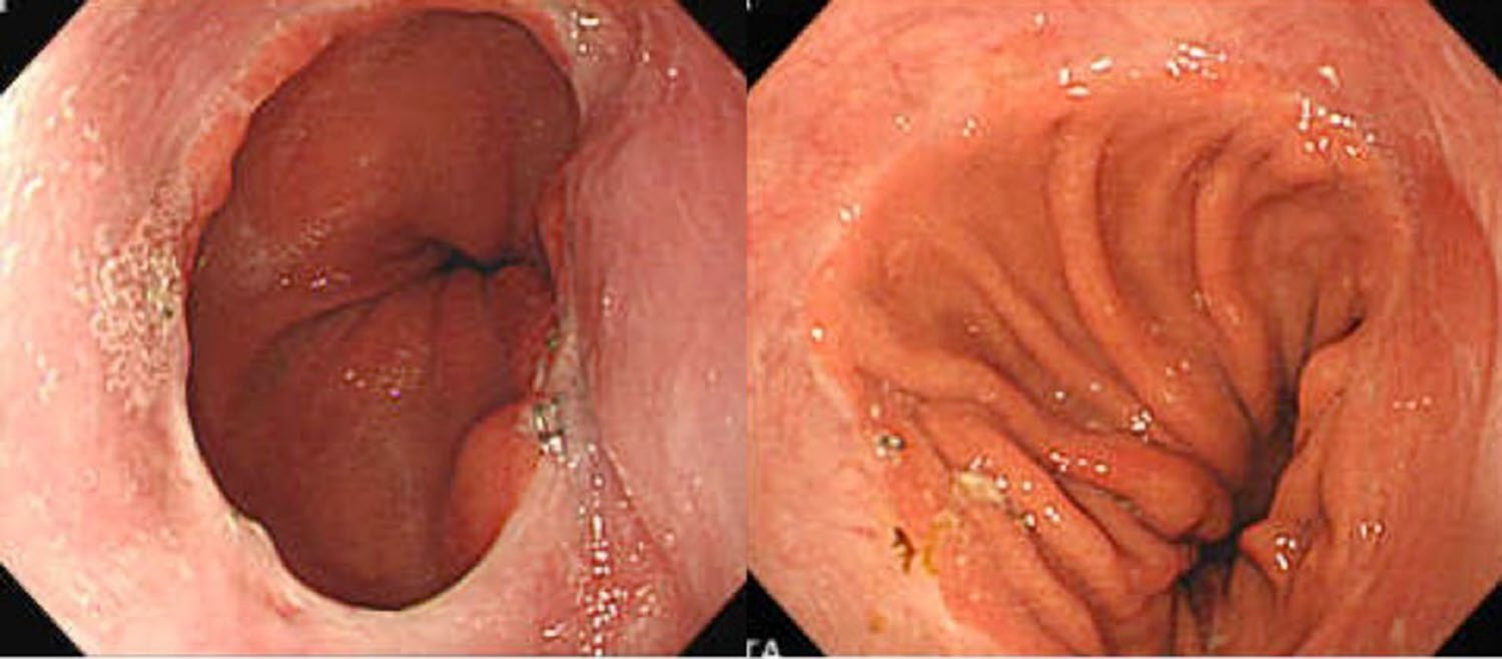
Postoperative endoscopic view of the triple-stapled quadrilateral anastomosis. The lumen is wide and quadrilateral in shape, 2 of the 4 sides are everted, and no mucosal defects are seen

Upon completion of thoracoscopic esophagectomy with mediastinal lymph node dissection, the patient is moved from the left lateral decubitus position to the supine position. Gastric mobilization with abdominal lymph node dissection and gastric conduit formation are then performed laparoscopically. A gastric conduit approximately 3 cm in diameter is made by dividing the stomach along the greater curvature with a linear stapler. The lesser curvature is resected starting at about 5 cm above the pylorus, and the right gastroepiploic vessels and 3 branches of the right gastric artery are preserved [9, 10]. After cervical lymph node dissection, the gastric conduit is pulled upward to the neck through the posterior mediastinum. The cervical esophagus has already been transected, and the gastric conduit is transected with an electronic scalpel about 5 cm below the proximal end. Before anastomosis, adequate mobilization of the cervical esophagus is needed to allow for placement of linear staples in the esophagus and stomach. The posterior walls of the esophagus and gastric conduit are placed in apposition, and 2 stitches are placed to anchor them (Figs. 1a, 2a). A 60-mm linear stapler (Endo-GIA60-3; Covidien) is positioned between the 2 stitches for construction of the V-shaped posterior wall of the anastomosis. [The anvil is placed in the esophagus, and the staple cartridge is placed in the stomach (Figs. 1b, 2b, c).] The anterior wall is then everted and elevated with the use of additional supporting sutures placed at regular intervals (Fig. 2d), and the anastomosis is closed with 2 60-mm linear staplers (Endo-GIA60-3; Covidien) (Figs. 1c, d, 2e, f). The most important feature of the TRIQ technique is that the anterior wall is closed in a gentle chevron-like shape with the use of 2 separate linear staplers, so the anastomosis takes on the shape of a wide quadrilateral. When the anterior wall is closed, it is important to avoid overlap between the two staple lines and the gastric conduit staple line. To avoid such overlap, we manually manipulate the tissue so that the staple lines do not intersect. To reduce tension at the convergence of the staple lines, a single-layer of sutures is placed at the points were the staple lines intersect, and the anterior wall is closed with Lembert sutures to reinforce the anastomosis and avoid formation of a tracheal fistula (Figs. 1e, 2g). The anastomosis is then wrapped with a greater omentum flap, and the omentum flap, esophageal wall, and gastric conduit wall are sutured (Figs. 1f, 2 h). The reconstructed gastric tube is then positioned orthotopically, and the cervical incision is closed in 2 layers over a vacuum drain. A postoperative endoscopic view of the reconstruction shows the wide oval-shaped anastomosis (Fig. 3 **)**.

## Results

Clinical characteristics of the 60 patients included in our study are shown in Table 1. The male:female sex ratio was 48:12, and median age was 67.8 years (range 48–81 years). Forty-three patients had undergone neoadjuvant chemotherapy. Neither the thoracoscopic or laparoscopic procedure was converted to open surgery in any patient. The tumor was located in the upper thoracic esophagus in 7 patients, middle thoracic esophagus in 34 patients, and lower thoracic esophagus in 19 patients. The pathological stages were as follows: stage 0 (*n* = 3), stage I (*n* = 6), stage II (*n* = 16), and stage III (*n* = 35). Operative and perioperative outcome variables are shown in Table 2. Median operation time was 474 min (range 320–680 min), and median blood loss volume was 104.4 mL (range 30–240 mL). Median postoperative hospital stay was 23 days (range 12–73 days). No Clavien-Dindo grade II–V anastomotic leakage was observed. Clavien-Dindo grade I anastomotic leakage occurred in 1 patient (1/60, 1.7%), and this leakage was resolved simply by delaying the patient’s oral intake. Anastomotic stricture did not develop in any patient.

**Table 1:** Clinical characteristics of the study patients (*n* = 60)

Age (years)	67.8 ± 8.3
Sex ratio (M/F)	48/12
Location of tumor	
Upper thorax	7
Middle thorax	34
Lower thorax	19
Neoadjuvant chemotherapy (∓)	17/43
Tumor pStage 0/I/II/III/IV	3/6/16/35/0
Pathology	
Squamous cell carcinoma	60
Adenocarcinoma	0

Mean ± SD value or number of patients is shown

**Table 2:** Operative and postoperative outcome variables (*n* = 60)

Operation time (min)	474 ± 78.4
Operative blood loss (mL)	104.4 ± 66.1
Postoperative hospital stay (days)	23 ± 12.8
Anastomotic leakage or stricture	
Anastomotic leakage	1 (1.7%)
Grade I	1
Grade II	0
Grade III	0
Anastomotic stricture	0

Mean ± SD values or number and percentage of patients are shown

## Discussion

Controversies remain regarding the surgical management of esophageal cancer. Unresolved questions include the optimal operative approach, extent of resection, role of multimodality therapy, and method of reconstruction following esophagectomy [11]. Minimally invasive esophagectomy techniques have been shown to be valuable alternatives to traditional open surgery. In our department, we treat all cases of esophageal cancer by means of combined thoracoscopic and laparoscopic esophagectomy, which is the least invasive approach. In a 2012 randomized trial conducted to compare minimally invasive esophagectomy and open esophagectomy, minimally invasive esophagectomy was shown to be oncologically equivalent to open surgery, and the short-term outcomes were favorable [12]. We have reported that thoracoscopic esophagectomy, in comparison to transthoracic esophagectomy, results in lower cytokine and polymorphonuclear leukocyte elastase production and thus causes less surgical trauma [13]. In this era of minimally invasive surgery, complications can spoil the benefits of the minimally invasive approaches and should be avoided as much as possible. For patients who undergo esophagectomy, anastomotic leakage or stricture can arise as either a short-term or long-term complication, and both negatively affect the patients’ quality of life. The frequent and still important problem of anastomotic failure after esophagectomy has stimulated development of a variety of anastomotic methods, with the optimum method still a matter of debate.

Hand-sewn anastomosis and stapled anastomosis (whether circular stapled or linear stapled anastomosis) are the 2 primary anastomotic techniques applied after esophagectomy. Hand sewing is the conventional method; it is both convenient and economical because it does not require the use of expensive specialized instruments. The disadvantage of hand-sewn anastomosis is that it takes a relatively long time to perform, and it requires surgical expertise, whereas the stapled anastomosis shortens the operation time and facilitates closure, especially in situations in which surgical visibility is poor. The circular stapler is useful for creating a stable anastomosis, but it is sometimes difficult to insert the anvil shaft of the circular stapler into the esophagus, and it is sometimes difficult to anastomose the esophagus and gastric conduit. These difficulties arise because manipulation of the stapler’s anvil in the esophagus is limited by the sternum and clavicle, and manipulation of the stapler’s center rod through the posterior wall of gastric conduit is challenging. The excessive traction on the esophagus and bending of the anastomosis that result from maneuvering the anvil shaft and center rod can compromise blood supply and put too much tension on the anastomotic site. These factors cause leakage and stricture [14]. The result of anastomotic complications was reported 0–26% (leakage) and 11–40% (stricture), so some reports have indicated that the esophagogastric anastomosis created with a circular stapler is not better than that created by hand sewing [11, 15–17].

Problems with hand-sewn anastomosis and circular-stapled anastomosis have led to linear stapling techniques [14, 18–20]. Collard and colleagues reported the terminalized semimechanical side-to side suture technique in 1998 [21]. This method allows for construction of a much larger anastomosis than the classic single-layer running manual suture technique allows, and it was reported to result in a lower anastomotic complication rate than that resulting from manual end-to-side anastomosis. A linear stapler is used across the gastric and esophageal walls, which are placed side by side, so as to create a V-shaped posterior opening between the 2 lumina. With this method, the anterior aspect of the anastomosis is hand sewn by placement of a classic running suture. This method, however, resulted in formation of a minute, blind fistula in the immediate paraesophageal soft tissue in 1 of the 16 reported patients (6.2%) and in dysphagia in another of the 16 patients (6.2%) [21]. There have been some modifications to the Collard method. Ercan and colleagues modified the Collard method by changing the stapler orientation (by placing the anvil in the esophagus and placing the staple cartridge in the stomach) and closing the anterior wall in a linear, transverse fashion by hand sewing [22]. They reported 96% freedom from cervical anastomotic leakage at 30 days and 59% freedom from all-cause anastomotic dilatation by 3 months. Orringer and colleagues reported a modification consisting of a mechanical, side-to-side anastomosis for which they used the anterior wall of the stomach and posterior wall of the esophagus [19]. Their technique can be considered a stapled and partially hand-sewn technique. Use of their technique in 114 patients resulted in clinically significant anastomotic leakage in 3 (2.7%) patients and the need for 1 or more outpatient anastomotic dilatations within the first 3 months after surgery in 39 (35%) patients. Thus, the modified techniques have resulted in low anastomotic leakage rates.

The reduced incidence of anastomotic leakage resulting from the modified techniques can be explained by the fact that the stapled anastomoses are more expedient and less traumatic to the tissue; that the lateral stay sutures reduce tension on the anastomosis without compromising the gastric conduit microcirculation; and/or that the linear stapler provides triple-layered staple construction that is less traumatic and more watertight [5]. Anastomotic stricture seems to remain an issue. A substantial number of reported patients have required anastomotic dilatation. According to a report by Price and colleagues, who analyzed outcomes of linear stapling, circular stapling, modified Collard, and hand-sewn anastomosis techniques, the incidence of stricture was lowest for the linear stapling technique [3].

Because none of the methods devised to date have been fully satisfactory, we modified and improved upon the Collard method by developing a new, all-stapled, side-to-side anastomosis, TRIQ. The modifications are as follows: (1) the anterior wall is closed with a linear stapler; (2) the anterior wall is closed in a gentle chevron shape with 2 separate linear staplers; (3) the anastomosis is wide and quadrilateral in shape; and (4) a greater omentum flap is wrapped around the anastomosis. To date, when we have applied TRIQ, no grade II–V Clavien-Dindo anastomotic leakage has been observed, and there has been no postoperative stricture. The stricture rates reported for all other stapled anastomoses have ranged from 0 to 9% [14, 23, 24]. Stapled anastomoses approximate the mucosa at 2 of 4 (TRIQ) sides or at 3 (delta-shaped anastomosis and triangulating stapling technique) sides of the triangle because 2 of the 4 or all 3 sides are anastomosed in an everted fashion, resulting in accurate mucosa-to mucosa apposition, which is considered good for healing. Noshiro and colleagues speculated that an extroverted anastomosis may be superior to an inverted anastomosis because applying the staples in an extroverted fashion could make it possible to avoid scaring along the staple lines, scarring that can expand intraluminally [23]. In addition, for TRIQ, we create a V-shaped posterior opening between the 2 lumina, and the anterior wall is closed in a gentle chevron-like shape with the use of 2 linear staplers. Consequently, a wide quadrilateral lumen is formed, and this lumen is larger than those obtained by means of all other stapling techniques. To obtain wide lumen, adequate mobilization of the proximal esophagus is needed. In TRIQ technique, 60-mm linear stapler is used to create a V-shaped posterior wall, but it is not always necessary that all length of staple is placed in esophagus and stomach. At least 35 mm length of esophagus and length of staple is needed to construct good posterior wall of the anastomosis and a larger lumen is obtained than the 34-mm circular stapler (maximal size of circular stapler).

For TRIQ, we always wrap a greater omentum flap around the anastomosis and suture the flap, the esophageal wall, and the gastric conduit wall. There have been reports of the effectiveness of the pedicled omental flap. Dai and colleagues reported a circular stapling technique and omental flap that yielded a fairly low leakage rate (0.8%) [25], and Yoshida and colleagues described a triangulating stapling technique and omental flap that yield a comparatively low leakage rate (7.0% with omentum flap vs. 18.9% without omentum flap) [26].

## Conclusion

From our retrospective evaluation, we conclude that TRIQ can be performed easily and safely. Favorable short-term results have been achieved to date. Further investigation that involves a larger number of patients, comparison of the technique with other anastomotic techniques, and evaluation of long-term outcomes is needed to confirm the benefit of TRIQ for reconstruction after esophagectomy.
